# Perspective: Opportunities in recalcitrant, rare and neglected tumors

**DOI:** 10.3892/or.2013.2581

**Published:** 2013-07-02

**Authors:** BEVERLY A. TEICHER

**Affiliations:** Molecular Pharmacology Branch, Developmental Therapeutics Program, National Cancer Institute, Rockville, MD 20852, USA

**Keywords:** pancreatic cancer, sarcoma small cell lung cancer, recalicitrant tumors, rare tumors, neglected tumors

## Abstract

The ‘Recalcitrant Cancer Research Act of 2012’ defines recalcitrant cancers as having a 5-year survival rate of <20% and estimated to cause the death of at least 30,000 individuals in the US each year. Cancers specifically mentioned in the act are lung and pancreatic cancers. In addition to recalcitrant tumors, rare tumors are often neglected in the drug discovery arena. Sarcomas are ~1% of cancers. The NCI Specialized Programs of Research Excellence (SPOREs) provide disease-focused cancer center grants specifically to accelerate the impact of laboratory research on the treatment of patients. There are 3 SPOREs focused on pancreatic cancer, 7 SPOREs focused on lung cancer and 1 SPORE focused on sarcoma. Through the Developmental Therapeutics Program (DTP), NCI maintains the infrastructure and expertise for the operation of cell-free and cell-based high-, medium- and low-throughput assays. The current effort is on sarcoma, SCLC and pancreatic lines. The DTP functional genomics laboratory provides molecular analyses including gene expression microarrays, exon arrays, microRNA arrays, multiplexing gene assays, plus others as tools to identify potential drug targets and to determine the role of selected genes in the mechanism(s) of drug action and cellular responses to stressors. The DTP tumor microenvironment laboratory focuses on the discovery of targets and the development of therapeutic strategies targeting the tumor microenvironment and physiological abnormalities of tumors resulting from environmental factors or alterations in metabolic enzymes. The DTP maintains a group focused on determining the mechanism(s) of action and identifying potential surrogate markers of activity for select compounds integrating proteomics, transcriptomics and molecular biology platforms. In conclusion, the NCI has active SPORE programs and an internal effort focused on recalcitrant, rare and neglected cancers which are generating data toward improving treatment of these difficult diseases.

The cancer research community has witnessed and participated in the recent grassroots efforts of advocacy groups lobbying the US Congress, the nation’s most visible bully pulpit, to provide focused research efforts directed toward improving patient survival of some of the most deadly cancers. The ‘Recalcitrant Cancer Research Act of 2012’ defines recalcitrant cancers as having a 5-year survival rate of <20% and estimated to cause the death of at least 30,000 individuals in the US each year. The cancers specifically mentioned in the act are lung and pancreatic cancers. Pancreatic cancer is the 5th leading cause of cancer death in the US. Over 33,000 newly diagnosed cases and the same number of deaths are expected in the US each year with a 5-year survival rate of 5%. The American Cancer Society estimated that there were 221,130 new cases of lung and bronchus cancer and 156,060 deaths in the year 2011. The most aggressive type of lung cancer is the small cell lung carcinoma. Approximately 31,000 new cases of small cell lung carcinoma were diagnosed in the US in 2011 (1,2). Small cell lung cancer tends to metastasize early in the course of the disease. Approximately 67% of small cell lung cancer patients have extensive disease at the time of diagnosis. Small cell lung cancer is usually treated with chemotherapy rather than with surgery. Median PFS for extensive disease is 5.5 months, while median overall survival averages 9–11 months and the 5-year survival in patients with extensive disease is 5% (3–5). In addition to recalcitrant tumors, rare tumors are often neglected in the drug discovery arena. Sarcoma are ~1% of cancers.

There are some similarities between pancreatic cancer, sarcoma and small cell lung cancer (5–7). Pancreatic cancer and small cell lung cancer patients often present with extensive disease, all three cancer types tend to have a major stromal component and all three have a high level of specific genetic alterations that are characteristic of the disease and difficult to target with drugs (6–10). The first line chemotherapy for pancreatic cancer, gemcitabine, was FDA approved for that indication in 1996. The most recent drug approved for pancreatic cancer was erlotinib in 2005 (9).

Small cell lung cancer (SCLC) frequently recurs after conventional cytotoxic chemotherapy. SCLC is a neuroendocrine subtype of lung cancer and affects >200,000 people worldwide every year. In the US, SCLC represents 13–15% of all lung cancer cases and is the most aggressive form of lung cancer with nearly as many deaths as diagnoses per year. Although initially a chemotherapy and radiation-sensitive disease, SCLC recurs rapidly (11). Although over the past 3 decades there have been >52 randomized SCLC phase 3 clinical trials testing varied cytotoxic therapies, there have been few improvements in SCLC treatment, with no significant changes in the standard of care (12). The combination of etoposide and cisplatin remains standard first-line therapy for SCLC. In 2003 topotecan became the only drug approved for treatment of patients with relapsed SCLC. From 1977 through 1992, 126 SCLC cell lines were established from 96 patients at the NCI-Navy Medical Oncology Branch (13). These patients comprised approximately one fourth of the 407 SCLC patients treated on prospective therapeutic clinical trials during the same time period. A proportion of tumor cell lines were established from previously untreated patients with both limited and extensive stage small cell lung cancer. MYC family DNA amplification was present in 16 of 44 (36%) tumor cell lines from previously treated patients compared to 7 of 52 (11%) of tumor cell lines from untreated SCLC patients. *MYC* DNA amplification in tumor cell lines established from patients previously treated with chemotherapy continued to be associated with shortened survival (13,14). The apoptosis related gene caspase-8 is frequently silenced in SCLC tumors and cell lines usually, but not always, by promoter methylation. In 34 SCLC lines (12 MYC amplified), the caspase-8 gene expression was frequently lost (79%). MYC amplification was present in 45% of SCLC cell lines, which had lost caspase-8 expression, but in none of the caspase-8 positive lines. There is also frequent loss of expression of CASP10, DR5, FAS and FASL in SCLC. The loss of expression of proapoptotic components was higher in MYC amplified SCLC cell lines and these lines were completely resistant to TRAI (15). A SCLC subset is dependent on activation of Hedgehog signaling, an embryonic pathway implicated in development, morphogenesis and the regulation of stem cell fates (16,17). SCLC has a unique biology with frequent specific molecular and cellular changes. SCLC has unique chromosomal changes; dysregulation of tumor suppressor genes, oncogenes, and signaling pathways; and active early development pathways (18).

Sarcoma includes widely varied tumors now divided into types and subtypes. Sarcoma occur in patients of all ages with frequency spread evenly over the human age range. Sarcomas represent a heterogeneous group of cancers from soft tissue (muscles and fat tissue), bone, cartilage, peripheral nerve sheets, or from other connective tissues. Many of these tumors affect children and young adults accounting for 15% of all pediatric cancers. The incidence for individual sarcoma types is rather low and all combined amount to ~13,000 new cases per year (1). The estimated death rate for 2011 is ~4,500 patients. Although the specific cell of origin of many sarcoma remains unclear, sarcoma are all tumors of mesenchymal origin. The mesenchymal stem cell, a pluripotent cell, which gives rise to varied differentiated cells including osteocytes, adipocytes, chondrocytes, muscle cells, fibroblasts, neural cells and stromal cells, maybe the ultimate cell of origin for sarcoma. When mesenchymal stem cell genetics go wrong and malignant transformation occurs sarcoma including osteosarcoma, Ewing’s sarcoma, chondrosarcoma, rhabdomyosarcoma, synovial sarcoma, fibrosarcoma, liposarcoma and many others can initiate. Our knowledge of sarcoma genetics is increasing rapidly. Two general groups, sarcoma arising from chromosomal translocations and sarcoma with very complex genetics, can be defined. Genes that are frequently mutated in sarcoma include TP53, NF1, PIK3CA, HDAC1, IDH1 and 2, KDR, KIT and MED12. Genes that are frequently amplified in sarcoma include CDK4, YEATS4, HMGA2, MDM2, JUN, DNM3, FLT4, MYCN, MAP3K5, GLI1 and the microRNAs miR-214 and miR-199a2. Genes that are upregulated in sarcoma include MUC4, CD24, FOXL1, ANGPTL2, HIF1a, MDK, cMET, TIMP-2, PRL, PCSK1, IGFR-1, TIE1, KDR, TEK, FLT1 and several microRNAs. While some alterations occur in specific subtypes of sarcoma, others cross several sarcoma types (10). Bone and soft tissue sarcoma are treated classic cytotoxic agents including vincristine, dacarbazine, doxorubicin, cyclophosphamide and cisplatin with more recent additions of gemcitabine and docetaxel. Discovering and developing new therapeutic approaches for these relentless diseases is critical. The detailed knowledge of sarcoma genetics may allow development of sarcoma subtype-targeted therapeutics.

The NCI Specialized Programs of Research Excellence (SPOREs) provide disease-focused cancer center grants specifically to accelerate the impact of laboratory research on the treatment of patients. There are currently 3 SPOREs focused on pancreatic cancer and 7 SPOREs focused on lung cancer. The first and only sarcoma SPORE so far was awarded in 2010 (http://trp.cancer.gov/). In addition, a component of NCI Developmental Therapeutics Program’s mission is to improve the treatment of recalcitrant, rare and neglected cancers through the discovery of potential therapeutic targets, the screening of new agents, the identification of genomic and epigenetic vulnerabilities, and the identification of potential therapeutic combinations using state-of-the-art drug discovery, molecular characterization, and mechanism-of-action techniques and through interactions collaboration with the cancer research community and other NCI laboratories. To achieve these goals, the NCI operates the classic NCI60 cell line screen, a target validation and screening laboratory, a functional genomics laboratory, a tumor microenvironment laboratory, a drug mechanism group, a cell characterization laboratory and a translational support laboratory. This effort is currently focused on sarcoma, small cell lung cancer and pancreatic cancer, and has collections of 70–80 human sarcoma cell lines, 70–80 human small cell lung cancer cell lines and 21 human pancreatic cancer cell lines.

New therapeutic approaches are needed to improve long-term survival in these diseases. The challenge of sarcoma is that it is 50–100 unique diseases, each with relatively small patient populations. Growing many types of sarcoma as xenografts has been difficult making *in vivo* proof of activity of agents in some types of sarcoma difficult. With SCLC the challenge is working with the cells in culture. SCLC grows as clusters making it difficult to get accurate cell counts at the beginning and conclusion of experiments. In clinical trial, the extreme aggressiveness of recurrent SCLC is a challenge in assessing the efficacy of new therapies. This program will provide a public database on the cell line panels including: i) gene/exon expression; ii) microRNA expression; iii) mutations; iv) subpopulations; v) response to standard and investigational anticancer agents; vi) protein expression and activated pathways; and vii) cell surface and intracellular markers. These data will be offered as a resource to the cancer research community. The panels and the characterization data will be used to identify targets and target combinations from which to generate new drug discovery endeavors at NCI and in the cancer research community. Currently, the sarcoma and SCLC lines are being screened in 9-point concentration response in triplicate to all of the FDA approved anticancer agents and to ~400 investigational agents, characterized genetically by exon array and microRNA array, characterized for cell surface protein expression and assessed for metabolic alterations. The drug discovery process is agnostic with respect to therapeutic approach using the best therapeutic strategy, small molecule or protein to approach each target.

NCI’s widely recognized classic 60-cell line screen has nine cancers, including leukemia, melanoma, non-small cell lung, colon, brain, ovary, breast, prostate and kidney (19–23). These cell lines were carefully selected for growth characteristics and reliability that were suitable for a 48-h compound exposure and continuous use. The NCI60-cell line screen has served the global cancer research community for >20 years. The aim of the screen is to identify agents (small molecules, proteins or other) showing growth inhibition or killing of tumor cell lines. The NCI60 screen has been conducted on a unique large database of compounds that allows the 60-cell line concentration response curve pattern produced by a given compound to be compared with response patterns of other compounds to identify similarities and potentially identify compound targets or mechanisms (COMPARE). The vision for the role of the NCI60 cell line panel has grown and broadened along with the technologies available to explore the cancer cell. There have been >200 peer-reviewed studies published on the NCI60 cell line panel over the past 5 years. While the majority of the reports are medicinal chemistry, drug response and compound studies (45%); many center on gene expression, genomics and development of gene signatures (24%). Other prominent topics include mutation analyses (7%), proteomics (6%), development of bioinformatics methods (4%), biomarkers (4%), microRNAs (3%), metabolomics, epigenetics and pathways analyses (1% each) (16–20). Cancer research community investigators both contribute and receive data from this resource. The NCI60 screen will continue to provide the cell lines, and RNA or DNA from the 60 cell lines to the cancer research community.

Through the Developmental Therapeutics Program (DTP), NCI maintains the infrastructure and expertise for the operation of cell-free and cell-based high-, medium- and low-throughput assays. The screening laboratory is equipped with extensive robotics, high content imaging, an Octet system for label-free quantitation of molecular (protein-protein and protein-small molecule) interactions, and maintains human sarcoma, SCLC and pancreatic cancer-focused cell line banks. This laboratory also has a BD FACSAria III flow cytometer, which is used to identify and map cell surface and intracellular markers and targets as well as to sort cell subpopulations. The current effort is to identify targets and characterize subpopulations within the sarcoma, SCLC and pancreatic lines. The FACSAriaIII is also used to assess response of cells to varied alterations in genes and response to exposure to varied chemical entities. The screening laboratory is a central focus for initiating projects and is the hub for carrying out screens with a goal to identify potential drug targets and unexpected sensitivities and to design screens to identify starting chemical matter for medicinal chemistry campaigns. A fragment library has been added to the compound screening sets to facilitate fragment-based drug design.

The Developmental Therapeutics Program functional genomics laboratory provides molecular analyses including gene expression microarrays, exon arrays, microRNA arrays, multiplexing gene assays, RNA interference, methylation assays, plus others as tools to identify potential drug targets and to determine the role of selected genes in the mechanism(s) of drug action and cellular responses to stressors (24–29). These methods are useful to probe for insights into the mechanism(s) of action of novel agents, as well as for detailed analyses of specific cells. The functional genomics laboratory is a resource that is widely tapped by collaborators within NCI as well as external investigators. This laboratory has taken on large-scale projects including profiling gene expression changes in the NCI60 cell lines following exposure to 15 anticancer agents with varied mechanisms of action after 2-, 6- and 24-h exposure at two concentrations. Analyses of these data may elucidate cellular responses to the drugs and potentially lead to new rationale drug combinations or elucidate new points of vulnerability in the malignant cells which can be targeted with therapeutic agents. The functional genomics laboratory is currently conducting a full genomic characterization (gene expression, exon expression, mutations, deletions, amplifications, microRNAs and other non-coding RNAs) for human sarcoma lines and SCLC lines with a goal of identifying potential drug targets in normal and mutant proteins.

The Developmental Therapeutics Program tumor microenvironment laboratory focuses on the discovery of targets and development of therapeutic strategies targeting the tumor microenvironment and physiological abnormalities of tumors resulting from environmental factors or alterations in metabolic enzymes (30–38). This laboratory is proficient in anchorage-dependent and -independent growth assays, western blot analysis, immunohistochemistry, ELISA assays, detection of protein-protein interactions and protein-DNA interactions, cytofluorimetric techniques, protein-DNA binding assays, RNA interference and metabolic assays. The tumor microenvironment laboratory has deep expertise in tumor physiology including hypoxia and angiogenesis and is involved in preclinical and early clinical studies (30–32). The goal is to understand how physiologic conditions in tumors, influence the mechanism of action of anticancer agents, and to discover therapeutics that may take advantage of the tumor microenvironment to improve treatment outcome (33–38). Hypoxia inducible factor (HIF) is critically involved in the tumor survival responses to conditions present in the tumor microenvironment and has been explored as a target for anticancer agents alone and in combination with antiangiogenic therapies. The tumor microenvironment laboratory is conducting drug discovery efforts targeting protein: protein interactions of factors such as oncogenes and transcription factors as well as continuing to work on tumor microenvironmental targets.

The Developmental Therapeutics Program maintains a group focused on determining the mechanism(s) of action and identifying potential surrogate markers of activity for select compounds integrating proteomics, transcriptomics and molecular biology platforms (39–47). The drug mechanism group applies varied technologies to each project, including several proteomics platforms such as quantitative proteomics/phosphoproteomics using stable isotope labeling by amino acids (SILAC), tissue, organellar, affinity, post-translational modification proteomics with quantitative labeling technologies (ex., TMT and O^18^/O^16^), 2D-PAGE, multiple reaction monitoring (MRM), and drug conjugate (e.g. GSH) and metabolite analysis. Compounds prioritized by the DTP Biologic Evaluation Committee and compounds from the DCTD NExT Discovery Projects are assessed in experiments directed toward identifying molecular target(s) and mechanism(s) of action.

The Developmental Therapeutics Program translational science laboratory contributes broadly to collaborative projects. For example, detailed cell-based studies including experiments with endothelial cells to assess antiangiogenic potential, cancer cell and normal cells to assess potential for therapeutic benefit that contribute to the evaluation of varied natural product and synthetic compounds are performed regularly (48–51; Hollingshead *et al*, unpublished data). In collaboration with the biological testing group, >2000 xenograft tissues collected were processed by snap-freezing, formalin-fixing and isolation of RNA and DNA in a study to provide a resource to the cancer research community. This laboratory conducts detailed combination studies in cell lines.

In conclusion, the NCI has active SPORE programs and an internal effort focused on recalcitrant, rare and neglected cancers which are generating data toward improving treatment of these difficult diseases.
